# The Naval M.O. in Action

**Published:** 1917-05-26

**Authors:** 


### THE NAVAL M.O. IN ACTION.

MUCH has been written of the Army surgeon and his work at the Front and in the hospitals. But the surgeon of the Senior Service has for the most remained obscure in the eye of the public, for he belongs to the Silent Navy. Yet throughout all the hostilities he has done magnificent work; during the Battle of Jutland many of them died at their posts. For duty is the supreme call, and the naval surgeon obeys it wholeheartedly and gladly, with no thought as to its difficulties and dangers.

In peace-time he, who is distinguished from fellow-officers by the significant red slip between the gold lace on his cuff, is not as a rule worked hard; for then the death-rate of the British Navy, excluding deaths from accidents, is only about two in a thousand men. Interesting cases are also rare, and some of the illnesses from which landsfolk suffer are practically unknown. In fact, to enable the naval surgeon to keep himself up to date, he is required by the King's Regulations for the government of His Majesty's Naval Service to take once in every three years courses of post-graduate study at a Metropolitan hospital for a period of three months. When thus requalifying himself in knowledge with the advances of medicine, the surgeon or M.O. gets full pay and all travelling expenses, and lodging and provision allowances, and has no longer to pay fees for each course. Other facilities are also given him to aid him in keeping up with the march of science and surgery.

Foresighted as well as thorough are the training and methods of our naval doctors. Successful candidates, immediately after passing, qualify in a course of practical instruction in naval hygiene at Haslar Naval Medical School, which is attached to that famous hospital of the Navy, near Portsmouth. Their studies include special instruction in the diseases peculiar to our foreign stations, ambulance drill, bullet and splinter wounds, and other branches of naval surgery; the use of the Röntgen-ray apparatus; serum therapeutics, and other special instructions. So thoroughly is the medical side of our Navy organised and trained that within four days from the declaration of war hospital ships had been fully equipped and staffed, and sent upon their way to join the Grand Fleet in the North Sea.

Excepting the small craft and light cruisers all British warships carry two or more surgeons, of whom the fleet-surgeon is the senior or medical officer in charge. Under his directions are the sick-berth or hospital, and the trained staff of sick-berth stewards and attendants. The sick-bay or berth is one of the most comforting places on board, and, resembling a small hospital ward fitted with swing cots, is placed in the most comfortable spot on the lower deck. Every morning at 9 A.M. the medical officer, sitting at a little table in a screened-off place, with his day-book before him, sees "patients," prospective and other.

But when the great vessel is going into action the surgeons and their assistants are posted at two distributing stations, one forward and one aft, below deck and behind the ship's thick armour. If there is time every one of the crew before action bathes, and changes into clean white underclothing, so as to lessen infection of their wounds. Many of the officers and men are required to be instructed proficiently in first-aid, and every gun crew is supplied with a bag of dressings, so that prompt attention may be given the wounded.

Immediately the buzzers call "Attention" and the bugle sounds "Action stations," the sick-bay staff and the "idlers" detailed to help them fit out the two medical stations. Stores and equipment are speedily taken below. The operating platform or table, all necessary instruments, bowlfuls of antiseptic, a medical chest and the emergency chest, and all requisites are laid ready in each of the medical stations. On the grim work beginning overhead, stretcher parties gather the wounded, whom they "strike down," or lower below, by cleverly devised methods to the medical stations, where the injuries are dressed. Then the casualty is removed to the sick-berth or the ward-room or other part of the vessel used for the time being as hospital.

For the naval medical officer there is no dressing station outside the zone of fire, or just on the edge of it. He is right in the middle of it. He hears the deep-throated guns, feels the thud of falling projectiles as they smash against the armour, and the ear-splitting crash of the huge shells; and soon there creep down to the medical station the irritating fumes. At any moment his ship may be sunk, or go up in smithereens on a volcano of blinding crimson and purple fire. Yet, despite all danger, he ever keeps a deft hand and a cool nerve, doing his best for the wounded who may be coming down in an appalling stream.

The sick-berth and its staff are entirely under his direction, and he is required to visit the sick at least twice a day, and oftener when necessary. In his care, too, is the health of the ship generally, water and milk to be analysed, and provisions bought in foreign ports to be examined and passed by him. When the captain, accompanied by the heads of departments, inspects the vessel and crew on Sunday and also on Thursday the surgeon-in-charge is one of the party. He has, too, many returns, reports, forms, etc., to make out and fill up, dealing with his sick and their illnesses, certificates of "wounds and hurts" to give, and to keep a rough and fair journal of his practice, which has to be forwarded annually to the Medical Director-General of the Navy; in addition to forwarding quarterly a nosological return of the state of his patients and the ship's health in general. Every morning at 9 A.M. he must submit the sick-list to the commanding officer, in addition to rendering a weekly return of patients. No fewer than eighteen pages of The King's Regulations and Admiralty Instructions are crammed with details concerning the duties of the medical officer-in-charge. Yet, notwithstanding, the "doctor" or "chemist" or "Sawbones" in the piping years of peace has perhaps a more than usually easy time. But the war has proved the unprecedented efficiency of the Naval Medical Service.

During the Battle of Jutland they did supremely magnificent work, and many of them went down at their posts. On board one great vessel, upon which the Germans poured a heavy fire of gas and other shells, smoke and fumes filled the operating stations crowded with wounded, adding suffocation to the other dangers. The vessel staggered in a rough seaway, she shook under the pounding blows upon her hull and from the concussions of her guns. Cool and ready amid the dazing pandemonium and the reeling of the huge hull, the surgeons hurriedly slipped on gas masks, and carried on with the moaning casualties as adequately as possible. Another ship was holed, and her electric light cut off. The vessel, making water, was sinking quickly. The operating station was in darkness, and choking with the foul fumes of the gas shells. Yet here, single-handed, the surgeon toiled by the light of a lantern till at length he was ordered to clear the station, and get his wounded up on deck, for the ship was being abandoned. And of those in his charge not a life was lost.

